# Influence of stone load on the outcome of same-session flexible ureteroscopy for bilateral upper urinary tract stones: a multicenter retrospective study

**DOI:** 10.3389/fmed.2023.1163371

**Published:** 2023-11-15

**Authors:** Wei-Chao Tu, Xin-Le Zhang, Jun Wang, Bao-Xing Huang, Ding-Guo Zhang, Da-Wei Wang

**Affiliations:** ^1^Department of Urology, Ruijin Hospital, Shanghai Jiao Tong University School of Medicine, Shanghai, China; ^2^Department of Urology, Kunshan Integrated Traditional Chinese and Western Medicine Hospital, Suzhou, Jiangsu, China; ^3^Department of Urology, Shanghai General Hospital, Shanghai Jiao Tong University School of Medicine, Shanghai, China; ^4^Department of Urology, Shanghai Pudong New Area People's Hospital, Shanghai, China

**Keywords:** bilateral, flexible ureteroscopy, holmium laser, upper urinary tract stone, stone-free rate

## Abstract

**Purpose:**

This study aimed to evaluate the efficacy and safety of same-session flexible ureteroscopy (fURS) for the treatment of bilateral upper urinary tract stones and to examine the influence of stone load on the outcome of same-session fURS, stratifying by total diameter of stones (TDS) ≤30 mm vs. >30 mm.

**Patients and methods:**

We retrospectively reviewed all cases of same-session fURS performed for bilateral upper urinary tract stones at four institutions between January 2017 and September 2020. All patients were divided into two groups based on TDS, ≤30 mm and >30 mm. Data on patient demographics, stone characteristics, surgical results, and complications were collected and analyzed for differences between the two groups. Stone-free rate (SFR) was defined as patients endoscopically stone-free or with radiological fragments <2 mm of each renal unit.

**Results:**

A total of 121 patients with bilateral upper urinary tract stones underwent same-session fURS, consisting of 73 patients in the TDS ≤ 30 mm group and 48 patients in the TDS > 30 mm group. The mean bilateral stone size was 28.2 ± 12.2 mm (range: 9.1–38.4 mm), with a mean operating time of 97.1 ± 39.6 min (range: 19–220 min). The SFR was 54.5% after the first fURS, and SFR increased to 97.5% after re-fURS for residual stones. The operation time for the TDS > 30 mm group was longer than that of the TDS ≤ 30 mm group (85.1 ± 36.5 vs. 115.4 ± 37.4 min, *p* < 0.001). The SFR after the first fURS was significantly lower in the TDS > 30 mm group than in the TDS ≤ 30 mm group (25.0% vs. 73.9%, *p* < 0.001). Although there was no statistically significant difference in overall SFR between the two groups (93.7% vs. 100%, *p* = 0.060), the rate of re-fURS for residual stones was higher in the TDS > 30 mm group than in the TDS ≤ 30 mm group (75% vs. 26%, *p* < 0.001). There were no significant differences in length of hospital stay (LOS) (2.2 ± 0.7 vs. 2.3 ± 1.0, *p* = 0.329) or complication rate (10.9% vs. 14.6%, *p* = 0.582) between the two groups.

**Conclusion:**

The results suggested that same-session fURS can be effectively performed with a low complication rate. A higher SFR after the first fURS can be achieved in the case of bilateral upper urinary tract stones with TDS ≤ 30 mm, and priority should be given to same-session fURS.

## Introduction

There has been a notable increase in the use of fURS in the new “Stone Age”, and fURS is a well-tolerated and effective surgical therapy for urolithiasis (1, 2). The extensive use of fURS can be attributed to the technological progress that has been achieved in terms of the digital flexible ureteroscope and holmium laser. The introduction of a digital flexible ureteroscope utilizing chip-on-the-tip technology with outstanding image quality and visibility has tremendously improved the effectiveness of fURS. Digital scopes demonstrate shorter operation times due to the improvement in imaging quality (3, 4). Furthermore, the improvement of fURS technology has made it possible to treat bilateral urolithiasis at the same time.

Same-session endourological surgery for bilateral urolithiasis has attracted increased attention, and there is a growing body of evidence to suggest that same-session URS/fURS or PCNL is safe (5, 6). The advantages of same-session therapy for bilateral urolithiasis are as follows: single anesthetic, reduced length of treatment cycle, and cost savings, which appeal to both surgeons and patients (7). Although some publications have reported on and reviewed the safety and feasibility of bilateral same-session endourological management of stones, studies on same-session fURS for the treatment of bilateral upper urinary tract stones are still scarce, and there is no consensus on the effect of bilateral stone load on stone clearance rate or on the need for re-fURS due to residual stones. We still need to explore whether same-session fURS for bilateral stones is safe and has a high stone clearance rate in order to avoid secondary fURS for patients with bilateral stones. The aim of this study was to further extend the assessment of efficacy and safety of same-session fURS to the treatment of bilateral renal and/or ureteric calculi and to examine the effect of stone load on stone clearance rate.

## Materials and methods

### Ethics statement

This study was performed in compliance with the Declaration of Helsinki and according to the protocol approved by the Medical Ethics Committee of Ruijin Hospital, affiliated with Shanghai Jiao Tong University School of Medicine. All participants were informed of the study and gave informed consent.

### Patients

The records of patients who underwent same-session fURS for bilateral upper urinary tract stones at four centers (Ruijin Hospital, Shanghai General Hospital, Shanghai Pudong New Area People's Hospital, and Kunshan Integrated Traditional Chinese and Western Medicine Hospital) from January 2017 to September 2020 were retrospectively reviewed. Patients were included if they had a renal stone and/or upper ureteral stone located on the superior aspect of the fourth lumbar vertebra and a unilateral stone load of ≤ 20 mm. Preoperative evaluation included serum creatinine, urine analysis, and culture. Patients received antibiotics if the preoperative urine culture sensitivity test was positive, and the treatment continued until the culture result turned negative. Patients with acute renal failure due to obstruction of the bilateral upper urinary tract were excluded. The maximum diameter of the stone based on non-contrast CT (NCCT) was taken to represent stone load; in the case of multiple stones, the sum of all stone diameters is referred to as the total diameter of stones (TDS).

Ureteral dilation was performed by inserting double-J stents bilaterally (6Fr, Cook) for 1–2 weeks before fURS under local anesthesia to decrease the failure rate of placing the ureteral access sheath (UAS) and to avoid ureteral injury.

### Techniques and instruments

The operations were carried out by experienced surgeons under general anesthesia. Ureteral calculi and renal calculi on the smaller side were prioritized for treatment. A digital flexible ureteroscope (Storz FLEX-X^C^, Karl Storz, Germany) was advanced through a 12/14Fr UAS. The stone was fragmented with a holmium YAG laser (Lumenis, PowerSuite 60W, USA) under the 200-μm laser fiber setting at 0.6–1.5 J × 20–40 Hz. The surgeon decided between dusting and basketing according to stone size, hardness, location, and intrarenal and/or ureteral anatomy. Stone fragments of >2 mm were retrieved with a nitinol basket (N-Gage Nitinol Stone Extractor NGE-017115-MB; Cook Medical). The double-J stents were inserted bilaterally *in situ* in all patients. We defined stone clearance as being endoscopically stone-free or with radiological fragments of ≤ 2 mm in diameter based NCCT and complete removal of stones on both sides. The first assessment of SFR was performed in the second week after fURS. Patients who had residual fragments of > 2 mm were scheduled for re-fURS. The double-J stents were removed in stone-clear patients. The second assessment of SFR was carried out the day after re-fURS. The double-J stent was no longer inserted in cases of re-fURS. Intraoperative and postoperative complications were recorded.

### Statistical analysis

Statistical analysis was performed using SPSS 22.0. Data are expressed in the form mean ± SD. The Student's *t*-test was used for parametric data comparisons, and the rank-sum test was used for non-parametric data comparisons. *P* < 0.05 was considered to be the threshold for statistical significance.

## Results

A total of 121 patients underwent bilateral same-session fURS in our study, consisting of 91 men and 30 women. The mean age was 48.2 ± 12.9 years. The mean TDS was 28.2 ± 12.2 mm. Detailed clinical data on patients are listed in Table 1. The mean operative time and length of hospital stay (LOS) were 97.1 ± 39.6 min and 2.2 ± 0.8 days, respectively. The SFR was 54.5% (66/121) at the second-week follow-up after the first fURS, and the overall SFR increased to 97.5% (118/121) after re-fURS in the case of a residual stone >2 mm. As shown in Table 2, complications according to the modified Clavien–Dindo classification were observed in 15 patients (12.3%), consisting of 9.1% (11/121) Clavien I and 3.3% (4/121) Clavien II complications. There were no complications graded Clavien III or higher. Patients were stratified into two groups according to stone load: a TDS ≤ 30 mm group and a TDS>30 mm group. The two groups were similar in terms of gender, BMI, preoperative urine culture, and stone location. Detailed clinical data on patients in the two groups are listed in Table 3. The results indicated that higher stone load was associated with longer operation time (85.1 ± 36.5 min *vs*. 115.4 ± 37.4 min; *p* < 0.001) and a lower SFR at the second-week follow-up after the first fURS (7.9% vs. 25.0%; *p* < 0.001). The SFR in the two groups after re-fURS was similar (100% vs. 93.7%; *p* = 0.06), but the TDS > 30 mm group had a higher rate of re-fURS for residual stones than the TDS ≤ 30 mm group (75% vs. 26%, *p* < 0.001). No significant difference between the two groups was observed in the complication rate (10.9% vs. 14.6%, *p* = 0.582) or LOS (2.2 ± 0.7 vs. 2.3 ± 1.0, *p* = 0.329).

**Table 1:** Descriptive patient data.

**Variable**	**Value**
Patients, *n*	121
Age, years (range)	48.2 ± 12.9 (23–76)
Body mass index, kg/m^2^	24.8 ± 3.4
**Gender**, ***n***	
Male	91
Female	30
Positive preoperative urine culture, *n* (%)	8 (6.6)
**Distribution of stones**, ***n*** **(%)**	
Renal and renal	65 (53.7)
Ureteric and ureteric	6 (5.0)
Renal and/or ureteric	50 (41.3)
Proportion of lower calyx stones, n (%)	15 (12.4)
CT value, HU (range)	895.8± 185.6 (585–1,552)
Anticoagulants, n (%)	9 (7.4)
TDS, mm (range)	28.2 ± 12.2 (9.1–38.4)

TDS, Total diameter of stones.

P < 0.05 was considered to represent statistical significance.

**Table 2:** Surgical results and complications.

**Variable**	**Value**
Operation time, minutes (range)	97.1 ± 39.6 (19–220)
LOS, days (range)	2.2 ± 0.8 (1–6)
**SFR**, ***n*** **(%)**	
SFR after first fURS	66 (54.5)
SFR after re-fURS	118 (97.5)
re-fURS, *n* (%)	55 (45.5)
**Complications**, ***n*** **(%)**	
Clavien–Dindo I	11 (9.1)
Postoperative fever	3
Ureteral mucosal erosion without smooth muscle injury	3
Gross hematuria	5
Clavien–Dindo II	4 (3.3)
Pain requiring analgesia	1
Urinary tract infection/pyelonephritis	3
Clavien–Dindo III	0
Clavien–Dindo IV	0
Clavien–Dindo V	0

LOS, Length of hospital stay.

SFR, Stone-free rate.

fURS, Flexible ureteroscopy.

P < 0.05 was considered to represent statistical significance.

**Table 3:** Comparison between the two groups.

**Variable**	**TDS ≤ 30 mm**	**TDS > 30 mm**	***P*-value**
Patients, *n*	73	48	
Age, years (range)	46.0 ± 12.5 (23–76)	51.5 ± 13.0 (25–76)	0.220
Body mass index, kg/m^2^	25.1 ± 3.4	24.5 ± 3.3	0.317
**Gender**, ***n***			0.671
Male	56	35	
Female	17	13	
Positive preoperative urine culture, *n* (%)	3 (4.1)	5 (10.4)	0.262
Proportion of lower calyx stones, *n* (%)	9 (12.3)	6 (12.5)	0.999
CT value, HU (range)	890.5 ± 182.5 (585–1,552)	996.4 ± 192.8 (622–1,508)	0.262
Anticoagulants, *n* (%)	5 (6.8)	4 (8.3)	0.999
TDS, mm (range)	20.0 ± 5.1 (9.1-29.8)	34.6 ± 8.8 (30.1-38.4)	< 0.001
Operation time in first fURS, minutes (range)	85.1 ± 36.5 (22–200)	115.4 ± 37.4 (19–220)	< 0.001
LOS, days (range)	2.2 ± 0.7 (1–4)	2.3 ± 1.0 (1–6)	0.329
**SFR, n (%)**			
SFR after first fURS	54 (73.9)	12 (25)	< 0.001
SFR after re-fURS	73 (100)	45 (93.7)	0.060
re-fURS, *n* (%)	19 (26)	36 (75)	< 0.001
Complications, *n* (%)	8 (10.9)	7 (14.6)	0.582
Clavien–Dindo I	6 (8.2)	5 (10.4)	0.752
Postoperative fever	1	2	
Ureteral mucosal erosion without smooth muscle injury	2	1	
Gross hematuria	3	2	
Clavien–Dindo II	2 (2.7)	2 (4.2)	0.999
Pain requiring analgesia	1	0	
Urinary tract infection	1	2	
Clavien–Dindo III	0	0	
Clavien–Dindo IV	0	0	
Clavien–Dindo V	0	0	

TDS, Total diameter of stones.

SFR, Stone-free rate.

fURS, Flexible ureteroscopy.

LOS, Length of hospital stay.

P < 0.05 was considered to represent statistical significance.

## Discussion

Urolithiasis is a common medical condition across the world, with lifetime risk of stone formation ranging from 7 to 13% in North America and from 5 to 9% in Europe (8). Kidney stones affect ~1 in 11 people in the United States and approximately 1 in 17 adults in China (9, 10). In Asia, approximately 1%−19.1% of the population suffer from stones, with recurrence rates ranging from 21% to 53% after 3–5 years (11). The general prevalence of bilateral stones among this patient population has increased to 15%. Management of bilateral urolithiasis is challenging and complex, and the efficacy of same-session or staged treatment for bilateral urolithiasis remains controversial. Same-session interventions have been attempted in order to reduce the length of the patient's hospital length of stay, the loss of work days, the number of anesthesia sessions, and potential costs. However, whether or not bilateral urological surgery can be performed efficiently and safely is still in doubt, and the potential benefit of this challenging approach for patients remains ambiguous. Some publications have reported that same-session bilateral URS treatment of stones is safe and effective, and the safety is increased in centers with more fURS cases (6, 7, 12).

As URS/fURS is well tolerated and associated with a low complication rate, guidelines from both the European Association of Urology (EAU) and the American Urological Association/Endourological Society (AUA/ES) now recommend URS/fURS as the first-line therapeutic option for both ureteric and renal calculi (13, 14), Hence, reports on same-session fURS for treatment of bilateral urolithiasis have been on the rise in recent years. Rivera et al. reported that a majority of endourologists do not perform bilateral PCNL due to concerns for bilateral renal injury or bleeding, but would perform bilateral URS (5). The results of same-session fURS treatment are also affected by stone load, according to the published literature (15).

The number of reported same-session fURS cases is still small. Chon et al. reported on bilateral fURS for the first time in 2005 (16). Huang et al. performed same-session fURS in 25 bilateral renal stone patients, achieving an overall SFR of 70% after a single procedure and 92% after second and third procedures (17). In another study, Atis et al. reported on 42 bilateral renal stone patients who underwent same-session fURS; the SFR was 92.8% after the first procedure and 97.2% after the second (18). Alkan reported that the overall SFR was 88.6% after all procedures in 44 same-session fURS cases (19). These researchers all came to the same conclusion that same-session fURS is a safe and effective procedure that can be considered as a first-line treatment for bilateral renal stones in selected patients. The choice of same-session fURS for the treatment of bilateral urolithiasis in our study was dependent on many factors: patient demographics, stone size and location, insufficient treatment modalities (ESWL or PCNL), patient preferences, and the presence of obesity or coagulopathy.

Stone load is an important parameter that affects outcomes in fURS. Operation time was significantly longer for the TDS > 30 mm group than for the TDS < 30 mm group, with SFR after the first fURS of 25% and 73.9%, respectively; in other words, if the bilateral stone load is <30 mm, same-session fURS can avoid the need for a second fURS in 74% of cases. Overall, the SFR after re-fURS for residual stones was 100% in the TDS ≤ 30 mm group and 93% in the TDS > 30 mm group in our series. The rate of re-fURS was significantly higher in the TDS > 30 mm group than in the TDS ≤ 30 mm group. The SFR after the first fURS was only 25% in the TDS > 30 mm group, suggesting that we need to carefully select cases with bilateral stone loads >30 mm. There was no difference in LOS between the two groups. These results were statistically significant and consistent with the literature (20).

Particular attention should be paid to the complications of same-session fURS. Our data confirm that same-session fURS for bilateral urolithiasis is safe. The overall complication rate of same-session fURS in our study was 12.4%, which was consistent with prior studies. Compared with the TDS ≤ 30 mm group, there was no significant increase in complications in the TDS > 30 mm group, although operation time was prolonged in the TDS > 30 mm group. There were no major complications (Clavien ≥ III) in our study, but there were 15 minor complications (Clavien I–II), including three patients who had a postoperative fever over 38°C and received anti-pyretic treatment, with the fever fully resolved within 48 h. The incidence of postoperative infection did not increase in same-session fURS. The double-J stent was inserted for more than 6 weeks in three patients with ureteral mucosal injury, and no stenosis was found during follow-up. Pain requiring analgesia occurred in one patient; prolonged hematuria that resolved spontaneously occurred in five patients; and urinary tract infection occurred in three patients, who were treated with intravenous antibiotics.

The lower rate of complications in this study may be related to the placement of the pre-stent *in situ* 1–2 weeks before fURS and the routine insertion of double-J stents after fURS in all patients in order to avoid postoperative infection and acute renal failure caused by ureteral obstruction. Of course, pre-stenting in advance prolongs the treatment cycle and increases the patient's lower urinary tract irritation symptoms.

Our study has some limitations. Although our study represents the largest set of reported cases of same-session fURS from four centers and shows that this is an efficient and safe option in patients with bilateral stones, with a very good SFR, it was a retrospective study. Moreover, no comparisons were made with other treatments (URS/PCNL) used to treat bilateral stones. The success rates of any endourologic treatment are affected by stone-related, clinical, anatomic, and technical factors. For multiple stones, there are limitations in evaluating stone load based on stone diameter. Future studies should include large-scale, multi-center randomized controlled trials comparing same-session fURS and staged fURS. The selection criteria and outcome measures need to be standardized. Longer follow-up times are necessary to confirm the long-term value of same-session fURS.

Our research suggests that same-session fURS is a less invasive therapy that achieves a high SFR and acceptable complication rates in the management of bilateral renal and/or ureteric calculi in selected patients, especially for patients with TDS ≤ 30 mm. Stone load will affect the results of same-session FURS. SFR decreases when TDS is over 30 mm, and the rate of re-fURS also increases significantly. The indications for and uptake of same-session fURS are likely to rise in the future.

## Data availability statement

The raw data supporting the conclusions of this article will be made available by the authors, without undue reservation.

## Ethics statement

This research was reviewed and approved by the Ethics Committee at the Shanghai Jiaotong University Medical School Affiliated Ruijin Hospital. Informed consent was obtained from all individual participants included in the study. All procedures performed in this research involving human participants were in accordance with the ethical standards of the institution.

## Author contributions

W-CT, X-LZ, JW, and D-GZ: collection, assembly of data, and patient follow-up. B-XH: data analysis and interpretation. D-WW: conception, research design, and manuscript editing. All authors contributed to the article and approved the submitted version.
